# Anxiolytic and Antidepressant Use and Burnout: Optimism as a Mediator in Spanish Nurses

**DOI:** 10.3390/jcm10245741

**Published:** 2021-12-08

**Authors:** África Martos Martínez, Ana Belén Barragán Martín, José Jesús Gázquez Linares, María del Mar Molero Jurado, María del Mar Simón Márquez, María del Carmen Pérez-Fuentes

**Affiliations:** 1 Department of Psychology, Faculty of Psychology, University of Almería, 04120 Almería, Spain; amm521@ual.es (Á.M.M.); jlinares@ual.es (J.J.G.L.); mmj130@ual.es (M.d.M.M.J.); msm112@ual.es (M.d.M.S.M.); mpf421@ual.es (M.d.C.P.-F.); 2 Department of Psychology, Universidad Autónoma de Chile, Providencia 7500000, Chile; 3 Department of Psychology, Universidad Politécnica y Artística del Paraguay, Asunción 1628, Paraguay

**Keywords:** burnout, drug use, anxiolytics, antidepressants, optimism, nursing

## Abstract

The aim of this study was to analyze the relationship between burnout, the use of drugs (anxiolytics and antidepressants) and optimism in nurses. At the end of 2018, a cross-sectional descriptive study was carried out with a sample of actively employed nurses recruited by snowball sampling. The sample consisted of 1432 nurses in Andalusia (Spain), aged 22–58, who were working at the time of data collection, 83.2% of whom were women. Data were collected anonymously in an ad hoc questionnaire about sociodemographic information and use of anxiolytics and/or antidepressives: the Brief Burnout Questionnaire—Revised for Nurses (CBB-R) and the Life Orientation Test—Revised (LOT-R). Descriptive, mediation and moderation analyses were performed, with significant results having a *p*-value less than 0.05. The results on burnout showed significant relationships with use of the drugs. In particular, personal impact, job dissatisfaction and motivational abandonment were positively related to use of certain of the anxiolytics and antidepressants presented, while the correlation with the social climate was negative. Furthermore, optimism correlated negatively with drug use. Knowing that optimism can alleviate the repercussions of the use of drugs opens up new lines of research and the possibility of developing programs aimed at promoting a positive disposition in the face of complicated events.

## 1. Introduction

Burnout is defined in the International Classification of Diseases (ICD-11) as a severe problem that appears in response to chronic exposure to workplace stress that has not been successfully managed [1]. Its development has been linked to occupations that deal with others and its effects have a high cost, as they affect not only the workers themselves, but clients and organizations [2,3]. Nurses, who show higher burnout rates than the general population or even other healthcare professionals, are one of the groups most affected [4,5,6]. According to a recent meta-analysis, this syndrome is present in one tenth of the world’s nursing population, although the rate may be even higher, since workers who are the most “burnt out” end up leaving the profession or do not respond to prevalence surveys [6]. According to the nursing burnout syndrome factor model [7], when these professionals are burnt out, they feel that the climate among coworkers and supervisors is inadequate, they lose hope and enthusiasm for their work, and perceive the situation at work as affecting them personally. Thus, burnout impacts on organizations, because more nurses leave their job, performance is lower, there are economic losses and patient safety is endangered [8,9].

In the individual, burnout can have extensive psychological and somatic effects [10,11]. The association between depressive and anxiety disorders is especially significant [5,12,13,14]. According to Stelnicki et al. [5], nurses with clinically significant burnout have a higher likelihood of a stress-related mental disorder. These authors found that “burnt out” nurses were 17 times more likely to show posttraumatic stress disorder, 23 times more likely to develop panic disorder and 25 times more likely to develop generalized anxiety disorder than workers who were not burnt out. In this regard, the longitudinal study by Rudman et al. [15] showed that early development of burnout in nurses generates alterations in cognitive functions and insomnia more than a decade later.

In line with the above, Sang et al. [16] suggested that there is a trend to greater use of benzodiazepines in nursing, mainly for stress, and it is precisely stress which is one of the most decisive factors in developing the syndrome [17]. In addition, the risk of frequent use of this drug, which could be a dysfunctional means of coping with stress and exhaustion in order to avoid distress [18,19], increases when there are comorbidities with anxiety, depression and sleep disorders [16,20]. A study of human service workers showed that burnout is a risk factor for the use of antidepressants [21]. However, other studies have noted that, in spite of high vulnerability to developing mental alterations linked to stress, nurses show a lower tendency to seek help and treatment than the general population, especially for anxiety and depression [22,23]. This may be partly due to fear of diagnosis and shame, or concern for stigmatization and job loss [24]. Moreover, some studies have found a higher prevalence of use of these substances by nursing personnel than other healthcare workers (physicians, aides, technicians, etc.) [25], while other studies have identified them as those who use them the least [26]. This disparity could be due to several factors, among them the stigma associated with mental health among healthcare workers themselves [26]. So, there could be some subtreatment of both disorders in these workers [22]. Even so, anxiolytics, followed by antidepressants, are the most commonly used psychotropic drugs in Spain [27]. Moreover, similarities between exhaustion and depression and anxiety may lead to false diagnoses, resulting in erroneous treatment of people with this syndrome [13], especially in view of overlapping symptoms [28]. Rudman et al. [15] found significant associations between the presence of burnout at the beginning of one’s nursing career and depression over a decade later. However, once the current burnout, cognition and insomnia were controlled, this association disappeared, suggesting that overlapping symptoms impede proper diagnosis, even though they are different entities [29].

Personality is one of the individual variables linked with burnout that has been widely studied [30]. Nurses with personality profiles marked by neuroticism show the greatest likelihood of burnout [31], and they do not usually make use of motivational strategies to improve the work alliance [32]. On the contrary, optimism seems to be negatively associated with this syndrome [33]. Optimism is a tendency or proneness to a positive affective state which influences an individual’s interpretation of information and behavior [34]. Optimistic people perceive negative situations as caused by temporary external sources and positive situations as the result of permanent internal causes [35]. This trait has been associated with a better ability to cope adaptively with complicated situations [36,37], better moods [38], and less emotional and physical anguish [39]. Optimism has also been shown to be a negative predictor of medical visits, promoting positive expectations about current problems and reducing anguish [40], and a mediator in subjective wellbeing [41]. A study by Hirata et al. [42] identified negative relationships between the use of hypnosedatives in recent months and optimism in nursing students and residents. In line with this, with regard to their practice, increased stress in the workplace has been linked to an increase in depressive symptoms, while optimism has been associated with their decrease [43]. Even though research in the role of optimism on the effects of burnout is scant, based on the above, it could be expected that this dispositional trait would reduce dysfunctional coping mechanisms and the effects of exhaustion. Therefore, as far as we know, this study starts out from a complete lack of previous studies analyzing the direct relationship between burnout and drug use by nurses. This should not be confused with the consequences of burnout on such disorders as anxiety and depression in nurses, as this is a subject which has been well-covered in the literature. This study also examined an individual variable, optimism, whose positive effects on burnout and its negative consequences (in this case, drug use) has not been demonstrated to date. Optimism being an individual tendency [34], knowing about its involvement in burnout and its effects would enable us to find out more about what other modifiable variables could be trained to improve this syndrome and its consequences. For example, in nursing, this variable has been shown to be a variable promoting commitment and favorable attitudes towards work, as well as reduction of burnout [44,45]. In turn, proneness to optimism in nurses has been directly associated with social support [46].

All of the above emphasizes the importance of job burnout in mental health [47] and drug use by nurses [5]. In today’s society, healthcare services are highly valued, and nurses are considered vitally important human resources [48,49]. It is therefore a priority objective to enquire into the factors that mitigate the negative impact of burnout in healthcare environments, especially given the present situation in which healthcare workers around the world are under very high stress from COVID-19 [50,51], and in view of the negative consequences of drugs on their users and those who depend on their users’ care [52]. Therefore, the objective of this study is to analyze the relationship between burnout, drug use (anxiolytics and antidepressants) and optimism in nurses.

To date, many of the publications on burnout and the use of anxiolytics and antidepressants have not undertaken these problems in such a way that a relationship between them can be established. In most cases, variables that intervene one way or another are identified; one will find discussion, for example, of work climate as an organizational variable, low income as a socioeconomic variable or stress as an individual variable. The latter is presented as a variable intervening negatively, as the role of stress in a hypothetical model would be be associated with the negative effects that burnout has on the use of anxiolytics/antidepressants. In our case, in what is a novel contribution, optimism is included as an individual variable, its positive implications giving the model a new twist, contrasting with the consideration of personal variables previously proposed only in negative terms. Taking this approach, it would be possible to design an intervention based on an individual’s positive characteristics, which should be kept in mind in effective program designs.

## 2. Materials and Methods

### 2.1. Participants

The original sample consisted of 1548 nurses in Andalusia (Spain). Based on the review of the answers to a series of control questions distributed in the questionnaire, 60 were discarded due to incongruent or random answers. In addition, as burnout was the main variable in the study, 56 were eliminated because they were not actively employed at the time of data collection. Therefore, the study sample was *N* = 1432. Participant age was in a range of 22–58, with a mean of 30.86 (*SD* = 6.33), of whom 93.2% were women. With regard to marital status, 61.3% (*n* = 878) were single, 36.1% (*n* = 517) were married and the remaining 2.6% (*n* = 37) were either separated or divorced. With regard to employment situation, 76.1% (*n* = 1090) had temporary contracts and 23.9% (*n* = 342) had permanent contracts.

### 2.2. Instruments

In addition to sociodemographic data, the participants were asked about their use of various anxiolytics and depressants. Specifically, they were asked about the frequency with which they took some of the following ten psychotropic drugs in the past ten months: alprazolam, lorazepam, diazepam, clorazepate dipotassium, bromazepam, fluoxetin, paroxetin, citalopram, escitalopram and sertralina. These drugs were mentioned using their generic name as well as some of the better known brand names in parentheses to facilitate their recognition (e.g., “Lorazepam (Idalprem, Orfidal, Placinoral, etc.)”). The use data was first collected on a Likert-type scale (never, sometimes, often, routinely) and then recoded as (no/yes) to attain dichotomous use variables for each of the drugs.

The Brief Burnout Questionnaire—Revised for nurses (CBB-R) [7] was used to evaluate burnout. It consists of 15 items, answered on a five-point Likert-type scale, and has a four-factor structure: (1) Personal Impact (e.g., *I am rather fed up with my job in general*); (2) Job Dissatisfaction (e.g., *My current job lacks any interest*); (3) Social Climate (e.g., *The employees back each other at work*); and (4) Motivational Abandonment (e.g., *My professional work currently offers me few personal challenges*). In this study, reliability was adequate, with McDonald’s Omega coefficient *ω* = 0.81 and greatest lower bound (GLB) = 0.82 for Personal Impact, *ω* = 0.64 and GLB = 0.64 for Social Climate, *ω* = 0.68 and GLB = 0.70 for the Job Dissatisfaction scale, *ω* = 0.49 and GLB = 0.51 on the Motivational Abandonment dimension and *ω* = 0.84 and GLB = 0.88 on the total questionnaire.

The Spanish version [53] of the Life Orientation Test—Revised (LOT-R) [54] was also administered. Six items are oriented toward dispositional optimism, while the remaining four are considered fillers, that is, their function would be to make the test content less obvious. Of the content items, three are written positively (optimism) and three negatively (pessimism). In line with the original authors’ theoretical proposal, the optimism–pessimism construct could be considered unidimensional with two extremes, although most studies on the instrument defend a two-factor structure [55]. The reliability analysis showed *ω* = 0.71 and GLB = 0.77.

### 2.3. Procedure

A CAWI (Computer Aided Web Interviewing) survey was used for data collection. The survey was divided into three sections: in the first were questions on sociodemographic and job characteristics; the scond included validated questionnaires for evaluating burnout and optimism; and, lastly, the questions on the use of psychotropic drugs were added. At no point were questions on personal data included, to ensure the anonymity of the survey. Furthermore, the participants were informed that data processing would be global, preventing any sort of identification.

Participation was voluntary, and, before any questions, the first page gave information on the study and its purpose. Participants gave their informed consent by marking a box designated for the purpose, which then gave them access to the questionnaire. They were asked to answer truthfully, and the anonymity of their answers was guaranteed. The participants took from 10 to 15 min to fill in the whole survey. For the detection of random or incongruent answers, control questions were included in the questionnaire. This study was approved by the Almería Bioethics Committee (Ref.: UALBIO2017/011).

### 2.4. Data Analysis

SPSS version 24.0 for Windows (IBM Corp., released 2016, Armonk, NY, USA) [56] was used for data processing and analysis. Instrument reliability was determined following Ventura-León and Caycho [57] by estimating the McDonald [58] Omega coefficient and the greatest lower bound (GLB).

First, frequency analyses were performed to find out the distribution of the sample by drug use, and descriptive analyses and Pearson correlations were calculated to explore the associations between the variables in the study. Gender differences in use were tested with Welch’s *t*-test [59], appropriate when variance is unequal or when group sizes are unequal [60]. To test the hypothesis proposed on the mediating role of optimism, a latent mediation model was computed by SEM (structural equation modeling) using the DWLS (Diagonal Weighted Least Squares) method, specifying two paths for the impact of burnout (X) on anxiolytic/antidepressant drug use (Y): a direct effect and an indirect effect through disposition to optimism (M). The lavaan package [61], JASP version 0.14 (Amsterdam, The Netherlands) [62], was used for this.

The following indices were used to evaluate model fit: ratio chi-square/degrees of freedom (χ^2^/df), which is considered optimum at values <3 [63,64,65] and acceptable at <5 [66], the CFI (comparative fit index), TLI (Tucker–Lewis index) and GFI (goodness of fit index), which according to Hu and Bentler [67] must provide values >0.95 to be considered an optimum fit and >0.90 for an acceptable fit; and other indices, such as the RMSEA (root mean square error of approximation), in which values <0.06 are considered optimum and <0.08 acceptable.

Finally, to examine optimism as a moderator, a simple moderation analysis was performed. The SSPS PROCESS macro was used to compute the simple moderation models [68]. Bootstrapping was applied with coefficients estimated from 5000 bootstrap samples.

## 3. Results

### 3.1. Drug Use, Burnout and Optimism

**Figure 1:**
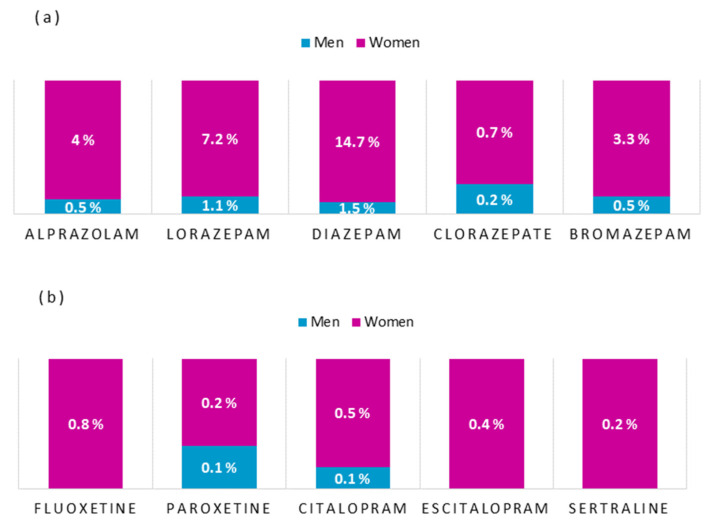
Percentages of (**a**) anxiolytic and (**b**) antidepressant use, by gender.

First, participants were asked about their use of five anxiolytics (Figure 1a) and five antidepressants (Figure 1b). The percentages of use in the total sample for each of the anxiolytics were: Alprazolam 4.5%, Lorazepam 8.3%, Diazepam 16.2%, Clorazepate 0.9% and Bromazepam 3.8%. Use of antidepressants in the sample was lower: Fluoxetine 0.8%, Paroxetine 0.3%, Citalopram 0.6%, Escitalopram 0.4% and Sertraline 0.2%. Figure 1 shows the use percentages for each of the drugs by gender.

The “use of anxiolytics” variable (constructed by counting all values except 0 = never on questionnaires) was significantly (*M* = 0.38, *SD* = 0.85) higher (*t*_Welch_ = −3.13, *p* < 0.01, *d* = −0.19) for women than for men (*M* = 0.24, *SD* = 0.59). “Use of antidepressants” did not significantly (*t*_Welch_ = −0.43, *p* = 0.666) differ between men (*M* = 0.03, *SD* = 0.28) and women (*M* = 0.04, *SD* = 0.28).

Table 1 shows the results of correlation analyses of the study variables and descriptive statistics. There were positive associations between anxiolytic and antidepressant use and the burnout dimensions of Personal Impact, Job Dissatisfaction and Motivational Abandonment. Social Climate was negatively correlated with use of anxiolytics and also with antidepressants. Optimism was negatively correlated with use of both types of drugs, but the association was stronger with anxiolytics.

### 3.2. Involvement of Optimism in the Relationship between Burnout and Use of Anxiolytics and Antidepressants

**Figure 2:**
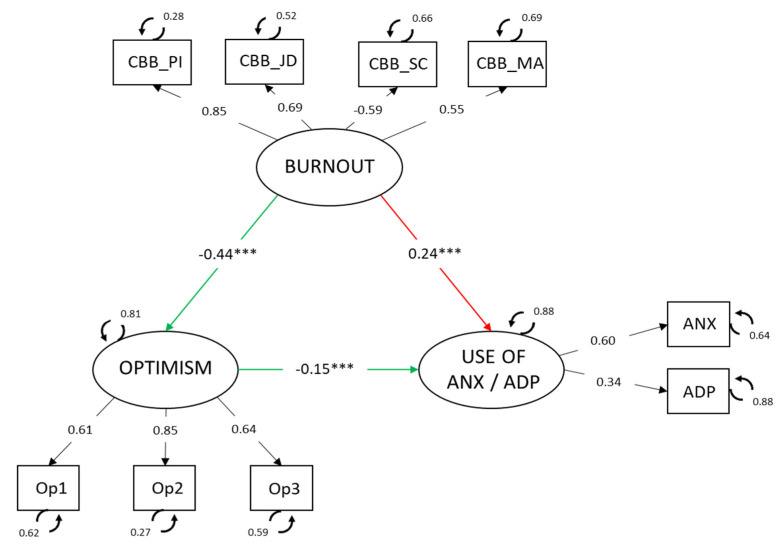
Latent mediation model. Standardized parameters are shown *** *p* < 0.001. Note: CBB_PI = Personal Impact; CBB_JD = Job Dissatisfaction; CBB_SC = Social Climate; CBB_MA = Motivational Abandonment; ANX = use of anxiolytics; ADP = use of antidepressants.

The hypothesized model (Figure 2) for use of anxiolytics and antidepressants showed adequate fit: χ^2^ (24) = 88.552, χ^2^/df = 3.69, *p* < 0.001, CFI = 0.99, TLI = 0.98, GFI = 0.99, RMSEA = 0.043 (CI 90% = 0.034, 0.053).

The relationships found between the latent variables in the model were: burnout related negatively to optimism (−0.44, *p* < 0.001) and positively to anxiolytic use (0.24, *p* < 0.001). The direct relationship between optimism and anxiolytics was also negative (−0.15, *p* < 0.001).

Furthermore, in view of the total effect of burnout on anxiolytic/antidepressant use 0.31, *p* < 0.001), and keeping in mind the magnitude of the indirect effect (0.07, *p* < 0.001), it may be concluded that the proportion (indirect/total) of this effect mediated by optimism is from 0.127 to 0.302.

Lastly, a simple moderation analysis was performed to test the interaction of different levels of optimism on the relationship between burnout and use of anxiolytics and antidepressants. The coefficients of the effects of each of the predictor and moderator variables were estimated, as well as the term of interaction on the dependent variable in each case (use of anxiolytics/antidepressants).

The results found in the models predicting use of anxiolytics reported significance only for the term of interaction Personal Impact * Optimism (*β* = −0.012, *p* < 0.01). The models for predicting use of antidepressants did not show any significance in any of the terms of interaction.

**Figure 3:**
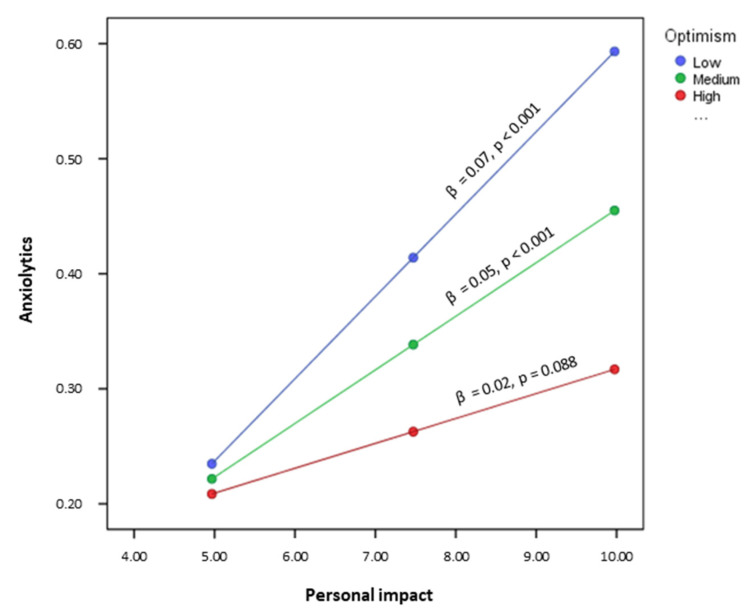
Interaction of “Personal Impact–Optimism” on the prediction of use of anxiolytics.

The prediction of Personal Impact on use of anxiolytics was estimated by Pick-a-Point for low, medium and high levels of optimism. This made it possible to find the conditional effect of the independent variable on the dependent variable at different moderator values. Thus, the results shown in Figure 3 suggest that the influence of the moderator variable occurred when it was low (*β* = 2.95, *p* < 0.001) or medium (*β* = 3.58, *p* < 0.001). Therefore, the effect of optimism as a moderator occurred when it was moderate.

The data found by applying the Johnson–Neyman technique show a wider range of values of the moderator and specify its involvement in the effect the independent variable exerts on the dependent variable. Therefore, Optimism acted as a moderator of use of anxiolytics when the score on Personal Impact was below 10.16 (approximately 88% of the participants).

## 4. Discussion

The objective of this study was to find out the relationship between burnout, optimism and drug use in nurses. The results showed, first, higher mean use of anxiolytics than antidepressants by nurses. It also showed that anxiolytics were used significantly more by women than men. These data are similar to those found in a previous study on nurses’ health behavior and gender, in which women showed a worse state of physical and mental health, and greater use of medication [20]. In addition, the literature shows an upward trend in the use of anxiolytics by nurses [16]. This is not surprising given the stress to which most of these workers are subjected [4,17,50].

Furthermore, a positive relationship was found between the use of antidepressants and anxiolytics and burnout’s personal impact and its effects in terms of job dissatisfaction and motivational abandonment. The relationship was negative between medication and the social climate dimension of burnout and optimism, especially with use of anxiolytics. In other words, greater use of tranquilizers and antidepressants by nurses is related to an absence of enjoyment and a lack of interest in their work, unhappiness at work interfering with nurses’ home life, a bad climate with coworkers and superiors and a generally less positive view of events. These results coincide with the literature showing burnout to be an agent involved in the development of mental alterations [10,11], especially depression and anxiety disorders [5,12,13,14]. It is also possible that, although there were no such comorbidities, overlapping symptoms, such as psychological distress, sleeping problems and problems concentrating, etc., could lead to erroneous diagnoses [15,28]. A negative association between the use of these medications and optimism could be due to the capacity of optimists for coping adaptively with adverse situations [36,37], considering the use of drugs as a maladaptive avoidance strategy [18,19]. Moreover, this trait promotes less physical and emotional anguish [39] and a more positive view of the future, which reduces visits to the doctor [40] and therefore medication.

A mediation model was also estimated to find out the role of optimism in the relationship between burnout and the use of anxiolytics and antidepressants. The findings showed negative relationships between burnout and optimism and a positive relationship between optimism and use of drugs, coinciding with previous studies [21,33]. Optimism was also confirmed as a variable acting on the relationship between burnout and the use of antidepressants and anxiolytics. That is, the presence of burnout in nurses affects drug use through optimism. Thus, workers who are exhausted by their work and who tend to see situations and the future hopefully would be less likely to use psychotropic drugs. People with an optimistic personality believe that negative situations are temporary and caused by external agents [35]; they are less distressed by emotionally overwhelming situations [39], such as extended exposure to stressful events in the workplace. This state of positive affect influences workers’ interpretation and behavior [34], so the worker perceives job distress as something temporary, and therefore would not need to have recourse to medical treatment to reduce the associated symptoms [38]. Travella and Parker [29] mentioned that professionals with burnout say that it has a specific extrinsic cause, so it could improve or be corrected if that cause were eliminated [29]. Optimistic individuals would not recur to medication because they see a way out of the situation.

Finally, the possible moderating effect of different levels of optimism on the relationship between the burnout variables and use of psychotropics was studied, showing that a medium or low level of optimism, present in over 80% of participants, increased the effect of the personal impact dimension of burnout on the use of anxiolytics. That is, the negative effect that the situation at work had on the personal life of nurses increased the use of anxiolytic medication when optimism was moderate or low. The pressure of distress and work stress on the nurses’ personal life is therefore related to an increase in the use of drugs to cope with anxiety and stress, but only when they have little hope. Furthermore, this personal impact could increase stress and generate sleep problems, which has been related to drug use [20].

In view of all of the above, we could say that the new findings of this study point to the existence of a direct relationship between the presence of burnout components and the use of antidepressants, especially anxiolytics, by nurses. We also found that optimism in nurses mediates the relationship between the variables mentioned. In this regard, it was demonstrated that optimism moderates the effect of burnout and psychotropic drug use. Thus, over 80% of nurses who show medium and low levels of optimism would consume more anxiolytics and depressants due to the impact that burnout has on their personal lives. In addition to the novelty of the findings, a further strength of this study is the representativeness of the sample due to the large number of nurses who participated in the study.

From this it may be inferred that policies directed at improving wellbeing and reducing stress in the workplace could be effective measures for reducing the use of anxiolytics and antidepressants, which are frequently used to reduce the distress caused by this syndrome. Previous works in the literature have shown that the use of psychotropic drugs by nurses has harmful consequences, not only for the employees themselves, but also when it comes to the performance of their duties. Therefore, measures for reducing their use should be taken into account for improving care quality and also employee quality of life. This study also showed that optimism is an ally against the use of psychotropic drugs in situations impacting personally on nurses from burnout. Specifically, this study considered the trait of optimism as a mediator between burnout and the use of psychotropic drugs. As this variable is a rather stable individual factor, we cannot be sure to what extent intervention would be beneficial. However, these findings could be directed at improving personnel selection or mental health surveillance. An interesting line for future research could determine whether state and trait optimism have similar effects. If so, it would be possible for healthcare management to establish measures for strengthening this variable and augmenting positive attitudes among healthcare workers, promoting, in turn, more effective coping with burnout. Intervention favoring a positive view of challenges that arise in their daily work could reduce drug use among nurses and possibly the appearance of other disorders associated with workplace anxiety and stress that have repercussions on mental health. As mentioned above, however, this hypothesis requires further study.

Before concluding, some limitations of this study should be mentioned. Firstly, data collection was carried out with self-report questionnaires and could therefore be affected by social desirability and subjective bias. Even though the questionnaire was completely anonymous, the potential stigma linked to admitting to drug use is not very likely to have been overcome. Therefore, this study may have underestimated the true magnitude of psychotropic drug use within the nursing population. Furthermore, there were no questions about the reasons for using psychotropic drugs. Therefore, we cannot be sure of the extent to which these drugs were taken for affections or for other reasons unrelated to exhaustion. However, although the study sample was representative and its results may be extrapolated to the general nursing population, in future, we believe that it would be a good idea to replicate the study in other units, such as psychiatric and mental health units, to identify the need for possible adjustments to the model. Likewise, it would be well to see if more objective measures of drug use could be used, such as data on medical attention or occupational health, selecting samples from clinical attention, and so forth. All of this would be directed at reducing the social desirability bias that can easily appear in self-report studies.

## 5. Conclusions

Nurses are highly valued because of their role in promoting social wellbeing. Burnout is one of the most common problems among these professionals, and a strong effort is being made by researchers to understand its triggering factors in order to prevent it. What happens, though, when it has already occurred? This study enquired into the effects that individual optimism has on the use of psychotropic drugs promoted by burnout. Although it would be ideal to promote individual, social and organizational measures that slow down the development of this syndrome, the truth is that the scarcity of these workers and the high demands that are made of them mean that its eradication seems very complicated at present. Therefore, knowing that a factor such as optimism can reduce recourse to anxiolytics and antidepressants opens new lines of research and the possibility of developing programs directed at promoting a positive disposition in the face of complicated events, frequent in nursing practice, by healthcare organizations. This would lessen the use of the psychotropic drugs most used in Spain, and although it has not been directly addressed in this study, could also affect the development of mental disorders linked to stress.

## Figures and Tables

**Table 1:** Correlation matrix.

		Personal Impact	Job Dissatisfaction	Social Climate	Motivational Abandonment	Optimism
Anxiolytics	Pearson’s *r*	0.190 ***	0.103 ***	−0.102 ***	0.094 ***	−0.138 ***
	95% CI Upper	0.239	0.154	−0.051	0.145	−0.086
	95% CI Lower	0.139	0.052	−0.153	0.042	−0.188
Antidepressant	Pearson’s *r*	0.110 ***	0.067 *	−0.074 **	0.084 **	−0.078 **
	95% CI Upper	0.161	0.118	−0.022	0.135	−0.026
	95% CI Lower	0.058	0.015	−0.125	0.032	−0.129
	M (SD)	7.47 (2.50)	8.43 (2.63)	12.2 (1.80)	9.12 (2.44)	8.25 (2.10)

Note. * *p* < 0.05, ** *p* < 0.01, *** *p* < 0.001.

## Data Availability

The data that support the findings of this study are available from the corresponding author upon reasonable request.
